# Biogas Production from Protein-Rich Biomass: Fed-Batch Anaerobic Fermentation of Casein and of Pig Blood and Associated Changes in Microbial Community Composition

**DOI:** 10.1371/journal.pone.0077265

**Published:** 2013-10-16

**Authors:** Etelka Kovács, Roland Wirth, Gergely Maróti, Zoltán Bagi, Gábor Rákhely, Kornél L. Kovács

**Affiliations:** 1 Department of Biotechnology, University of Szeged, Szeged, Hungary; 2 Institute of Biochemistry, Biological Research Center, Hungarian Academy of Sciences, Szeged, Hungary; 3 Institute of Biophysics, Biological Research Center, Hungarian Academy of Sciences, Szeged, Hungary; Oak Ridge National Laboratory, United States of America

## Abstract

It is generally accepted as a fact in the biogas technology that protein-rich biomass substrates should be avoided due to inevitable process inhibition. Substrate compositions with a low C/N ratio are considered difficult to handle and may lead to process failure, though protein-rich industrial waste products have outstanding biogas generation potential. This common belief has been challenged by using protein-rich substrates, i.e. casein and precipitated pig blood protein in laboratory scale continuously stirred mesophilic fed-batch biogas fermenters. Both substrates proved suitable for sustained biogas production (0.447 L CH_4_/g protein oDM, i.e. organic total solids) in high yield without any additives, following a period of adaptation of the microbial community. The apparent key limiting factors in the anaerobic degradation of these proteinaceous materials were the accumulation of ammonia and hydrogen sulfide. Changes in time in the composition of the microbiological community were determined by next-generation sequencing-based metagenomic analyses. Characteristic rearrangements of the biogas-producing community upon protein feeding and specific differences due to the individual protein substrates were recognized. The results clearly demonstrate that sustained biogas production is readily achievable, provided the system is well-characterized, understood and controlled. Biogas yields (0.45 L CH_4_/g oDM) significantly exceeding those of the commonly used agricultural substrates (0.25-0.28 L CH_4_/g oDM) were routinely obtained. The results amply reveal that these high-energy-content waste products can be converted to biogas, a renewable energy carrier with flexible uses that can replace fossil natural gas in its applications. Process control, with appropriate acclimation of the microbial community to the unusual substrate, is necessary. Metagenomic analysis of the microbial community by next-generation sequencing allows a precise determination of the alterations in the community composition in the course of the process.

## Introduction

Anaerobic digestion (AD) with concomitant biogas production is an environmentally attractive technology for the treatment of organic waste. Biogas provides environmental benefits with regard to waste treatment, pollution reduction, the production of CO_2_-neutral renewable energy and the improvement of agricultural practices through the recycling of plant nutrients [1].

Biogas can be burnt to produce heat, or combusted in gas engines for electricity generation, and after purification it can be used in any application for which fossil fuel natural gas is utilized today. AD is applied to a range of industrial waste streams, especially in the agro industry, which is a source of high concentrations of readily degradable organic material composed mainly of complex molecules. Despite the industrial-economic importance of the underlying microbiological events, little is known about the roles and activities of the microorganisms which inhabit the anaerobic niches [1]. 

In the European Union (EU), more than 7,800 biogas plants are currently operating, but none of them process primarily protein-rich waste, despite huge amount of such materials being generated continuously. These pollutants mount up in vast quantities and their disposal is costly and energy-consuming. The roughly 1 million tons of protein-rich waste produced annually by pig, cattle and bird breeding worldwide contains manure, blood and feathers [2], a biomass type classified as hazardous waste in several countries. The world’s broiler chicken production is growing faster than any other form of meat production. 30.9% of the world’s poultry meat is supplied by the EU [3]. The EU egg and poultry sector contributes 6.3% to the overall EU agricultural output, accounting for some €23.3 billion annually [4]. This means 500,000 tons of protein-rich waste per year in the EU alone. Many other parts of the world faces similar problem. 

Protein also accounts for a major part of the organic load in a dairy wastewater stream [5]. A low rate of AD, together with inhibition problems caused by certain components, have been identified as responsible for the restricted activity of biogas reactors operated with dairy effluent [6,7]. Other branches of the food industry, such as abattoirs, and the processing of whey, cheese, casein and certain vegetables, typically produce wastewater containing relatively high concentrations of protein [5]. Such forms of protein-rich biomass would be extremely valuable as biogas substrates if the basic questions concerning their AD could be solved. Besides the advantages of the biogas produced, the environmentally harmless fermentation residue is an excellent fertilizer for agriculture [2,6]. 

Proteins are composed of amino acids linked by peptide bonds, which are hydrolyzed by proteases upon decomposition. Amino acids are fermented via different pathways, depending on the nature and concentrations of the amino acids present [5,8]. The degradation products include short- or branched-chain organic acids, NH_3_, CO_2_ and H_2_. Amino acids are metabolized through two main routes: pairs of amino acids can be decomposed through the Stickland reaction; and single amino acids can be degraded in the presence of H_2_-utilizing bacteria [5,8]. The Stickland reaction is the simplest way to degrade amino acids and provides the cell with 0.5 mole of ATP per mole of amino acid transformed [9]. Stickland reactions are usually faster than uncoupled amino acid fermentation [5].

AD demands the concerted action of many groups of microbes, each performing their special role in the overall degradation process [10,11]. In the absence of terminal electron acceptors such as nitrate, oxygen or sulfate, the methanogenic conversion of organic matter is an essential feature of many ecosystems [12]. The optimal carbon/nitrogen/phosphorus (C/N/P) ratio for a high methane yield is around 100:3:1 [13,14]. The digestibility of carbohydrate-rich wastes can be improved by mixing them with substrates of high nitrogen content, thereby improving the C/N ratio [15-17]. In anaerobic fermentation, the acidogens and methanogens differ in their physiology, nutritional needs, growth kinetics and sensitivity to the environmental conditions [18,19]. Failure to sustain the balance between these two groups is the main cause of process instability [20].

The introduction of energy-rich [21] proteinaceous waste products in large quantities into the AD process is not recommended in view of the increased risk of inhibition by ammonia [6,10,22]. In the literature, the inhibitory level of the total ammonia concentration varies, depending on conditions such as the inoculum, the substrate, the acclimation need, the operation period, pH and temperature [23-27]. Free NH_3_ is the main cause of inhibition since it is membrane-permeable [23,28] and it causes a proton imbalance and/or a potassium deficiency [29-31]. The ammonium ion (NH_4_^+^) is less toxic [32]. Ammonia-adapted anaerobic consortia were inhibited at an NH_3_ concentration of 0.7-1.1 g NH_3_-N/L [25,27]. 

The acclimation of biogas-forming microbial communities to a wide variety of potentially inhibitory substances has been reported [33]. In the undisturbed natural microbial community, the inhibitory ammonia level can be as low as 0.08-0.10 g NH_4_^+^-N/L [28,34]. Nevertheless, a tolerance of up to 3-4 g NH_4_^+^-N/L for an adapted process has also been reported [25]. 

Edström et al. [35] used blood, stomach and intestinal content and food waste in co-fermentation with animal manure. Feedstock mixtures containing 8-15% of animal waste products could be co-digested under stable conditions at a total concentration of 4.5-5.0 g NH_4_^+^-N/L. Successful operation of anaerobic filters was achieved at 6.0-7.8 g N/L after an adaptation period [28,36]. Such experiments clearly demonstrate that it is possible to ferment with a concentration exceeding 4-5 g NH_4_^+^-N/L after an initial adaptation phase [33,37]. A lower biogas yield and/or CH_4_ yield was observed, however, in the case of an elevated ammonia load [38,39]. A common feature of all previous ammonia adaptation attempts was that the substrate with a high nitrogen content was fed together with a substantial amount of carbon-rich materials in order to approach the recommended C/N/P ratio. Relatively few studies have considered the effects of scaling-up in a biogas fermenter under stringently comparable conditions and reactor geometry [31]. 

NH_3_ has a deleterious influence on the acetate-utilizing methanogens; its effects on the H_2_-utilizing methanogenic Archaea and syntrophic bacteria are less pronounced [10]. Elevated ammonia levels (0.8-6.9 g NH_4_^+^-N/L) cause changes in microbial communities; the shift from aceticlastic methanogenesis to syntrophic acetate oxidation is a consequence of the effect of the inhibition by NH_3_ [40,41]. The relative number of some aceticlastic methanogens decreased in the ammonia-stressed reactor, whereas the abundance of bacteria increased. The ammonia tolerance of some syntrophic acetate-oxidizing bacteria such as *Clostridium ultunense*, *Syntrophaceticus schinkii* and *Tepidanaerobacter acetatoxidans* apparently bestowed a competitive advantage on the community [41]. Other studies revealed that members of the Methanosarcinaceae and Methanosaetaceae were strongly inhibited by NH_3_ [30,42].

A semiquantitative method has been developed for a rapid distinction between microbial taxa on the basis of their ammonia tolerance [43]. Archaea and bacteria grew similarly in the presence of 0.8-2.3 g NH_4_^+^-N/L. At ammonium concentrations of 4.4 g/L and 8.6 g/L, Archaea were quasi-absent or absent, respectively. In the AD microbial communities, the main competitors of the syntrophic acetate-oxidizing bacteria are the aceticlastic methanogens, including *Methanosarcina* and *Methanosaeta*. Aceticlastic methanogens are also known to be more sensitive than hydrogenotrophic methanogens to the levels of ammonia and volatile fatty acids (VFAs) [44].

The aim of the present study was to determine the possibility of applying protein-rich substrates as monosubstrates for the biogas-producing microbial consortium in laboratory-scale fed-batch operated anaerobic digesters. In order to test the limits of the system, in some experiments the reactors were fed with increasing amounts of protein-rich substrates until process failure took place. Conditions for sustainable, stable operation and scaling-up were also determined. The response of the microbial community to the protein substrates casein and pig blood was studied by measuring the process parameters and the changes in the microbial community composition, using a next-generation sequencing-based metagenomic approach.

## Results and Discussion

### Protease activity increases during growth on protein substrate

**Figure 1:**
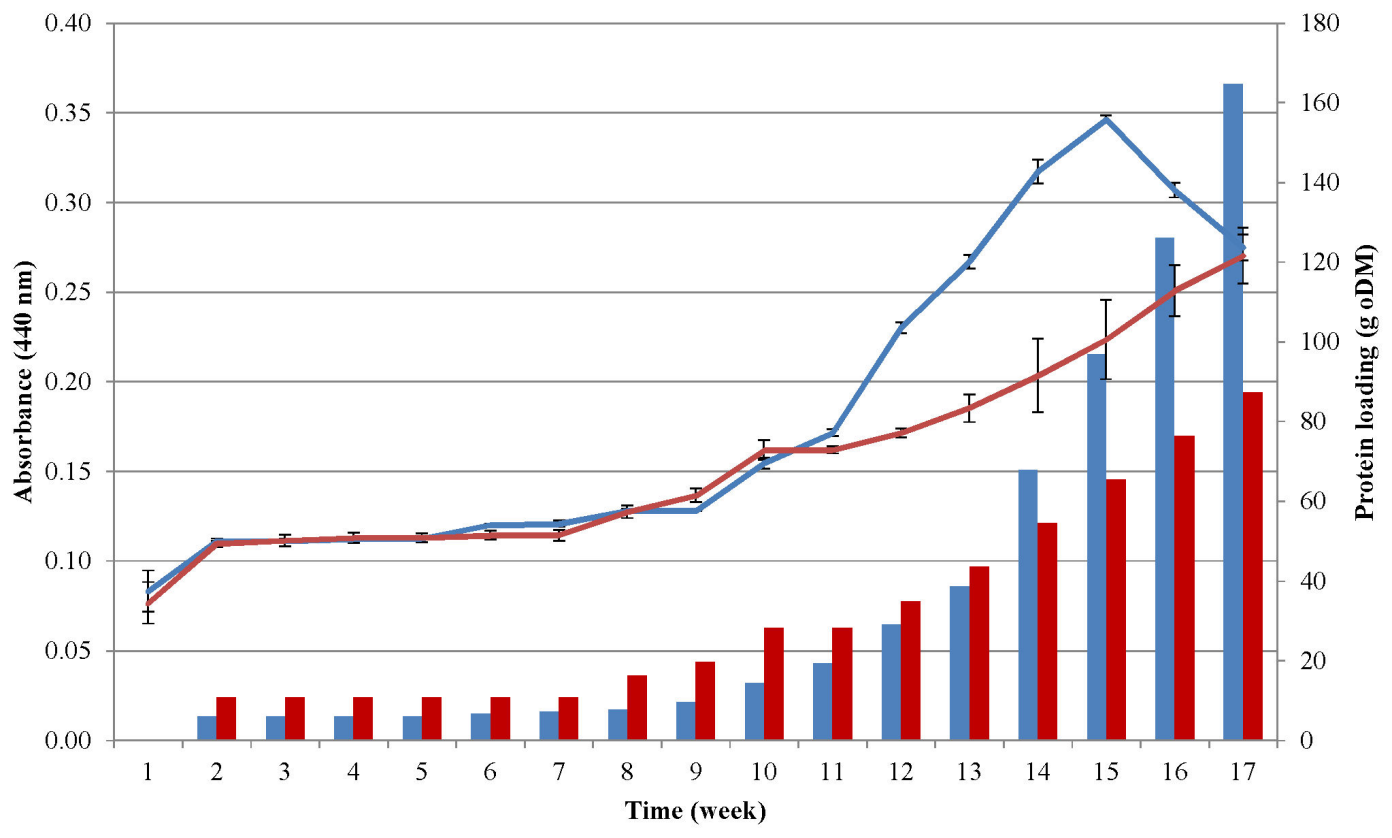
Protease activity changes during acclimation. The biogas-producing community was cultivated on casein (blue line) or pig blood (red line) as protein-rich substrates. The weekly protein doses are indicated by the blue (casein) and red (pig blood) columns. The vertical black line denotes the end of the adaptation period, i.e. feeding with a constant, low protein dose.

Decomposition of complex biopolymers to amino acids is a critical step, and one of the most important preconditions in the utilization of the protein-rich substrate is therefore the level of protease activity in the system. Hence, the protease activity was selected as the functional indicator of the performance of the microbial community during its acclimation to the unusual substrates. Figure 1 illustrates the increase in protease activity as the protein loading was elevated in the fed-batch AD process. Overall, the protease activity was augmented 5-fold in the case of casein and 3.5-fold when pig blood was the substrate relative to that at the start of the experiment, respectively. A single dose of protein was fed into the reactor every week and the maximum enzyme activity was found to be reached at 100 g of added casein, which is significantly higher than the protein load needed to produce the maximal biogas yield (Figure 1). This can be understood by taking into account the rise in the concentrations of volatile fatty acids (VFA), which had to be consumed by methanogens. Hydrolyzing bacteria are mainly responsible for protease production. For them the lower pH is not very disturbing, and they can survive at high VFA concentrations. The rate-limiting members of the biogas production process are the methanogens, whose growth and biological activity become restricted at high VFA and ammonia concentration (3-4 g N/L). 

Consequently, when the environment is still acceptable for the hydrolyzing bacteria and they produce proteases profusely, the same milieu becomes deteriorating for methanogens and biogas evolution. It is noteworthy that the protease activities and the rate of protein loading appear to correlate, i.e., a steeper increase of the protein loading was accompanied by a sharper rise in the total protease activity in the case of casein, whereas a less rapid elevation of the weekly protein dose was followed by a slower rise in the additional protease activity in the case of pig blood. The results indicate that enhanced protease activity is an important prerequisite for biogas production from a protein-rich biomass, but other factors may also play significant roles in the adaptation process and stabilization of the biogas-producing microbial consortium. 

### Biogas production from protein-rich substrates

**Figure 2:**
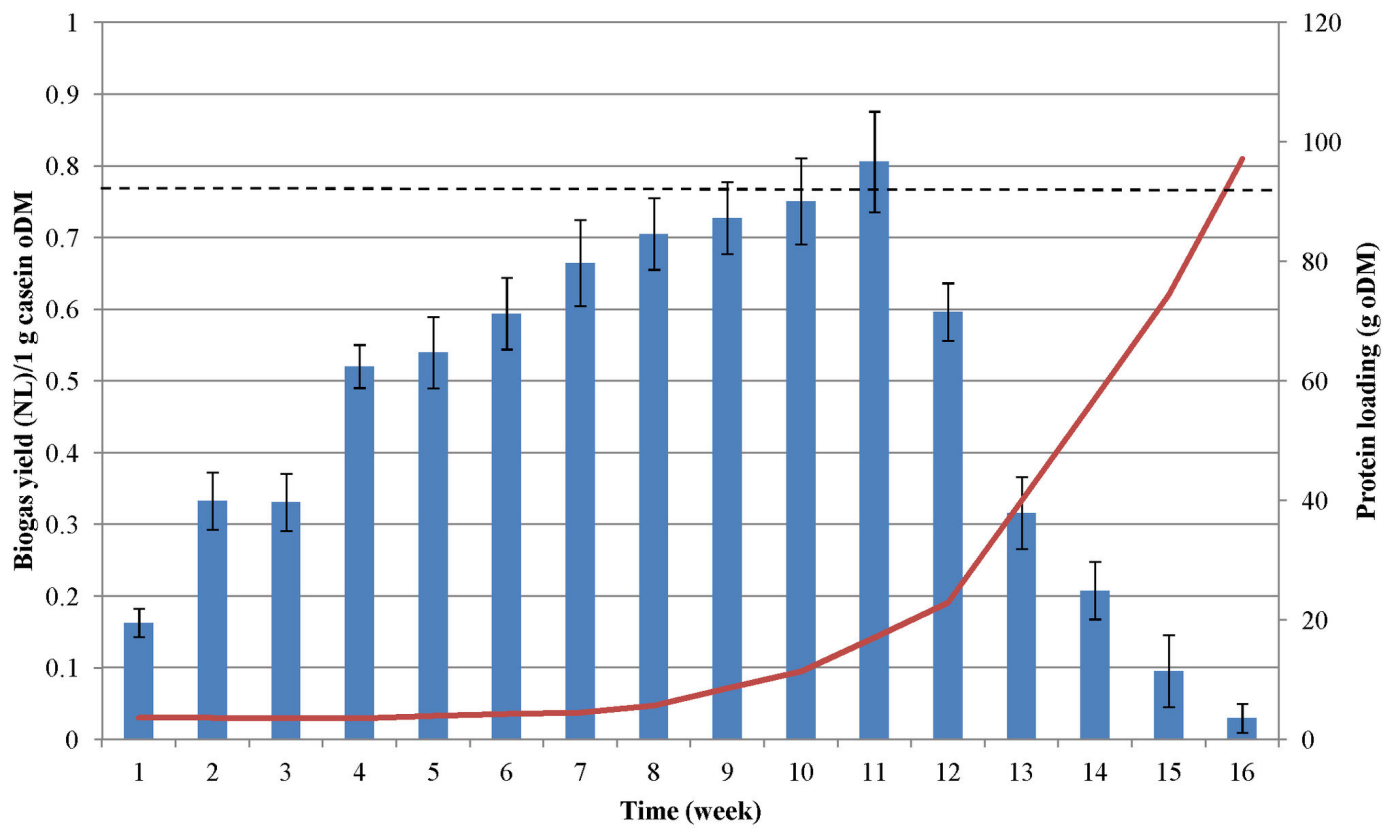
Biogas production from casein. The fed-batch reactors were fed with increasing amounts of protein at weekly intervals. The protein dosages are shown by red line. The columns indicate the average specific biogas yields generated each week. Dashed line indicates biogas productivity according to the batch test.

**Figure 3:**
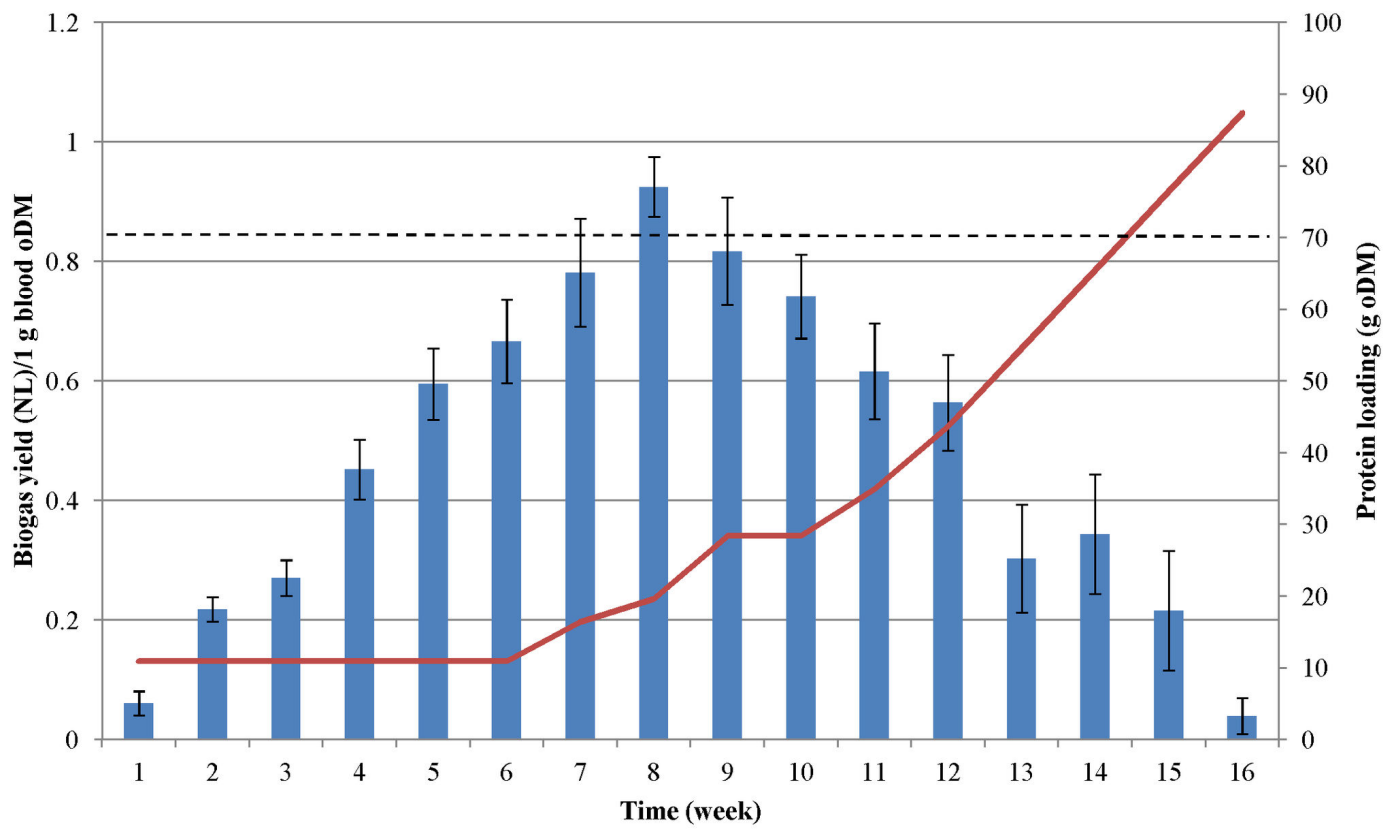
Biogas production from pig blood. The fed-batch reactors were fed with increasing amounts of protein at weekly intervals. The protein dosages are shown by red line. The columns indicate the specific biogas yield generated during each week. Dashed line indicates biogas productivity according to the batch test.

Biogas evolution started immediately after the reactors were supplied with the protein monosubstrate, i.e. before any change in the protease activity or process parameters was observable. This indicates that the community already included some microbes capable of degrading proteins, and during the acclimation period these gradually became dominant members of the community under the selection pressure of the feed composition. At the peak of biogas production from the protein-rich substrates (weeks 8-12), the microbial community generated 3.5 times more gas from a unit amount of protein than at the start of the adaptation. The maximum biogas yields were observed when the weekly load of casein was 30 g (Figure 2) and that of pig blood protein was 20 g (Figure 3). 

As the protein load was dispensed weekly, the daily biogas yield varied during the feeding period. One or two days after substrate addition, the biogas production increased rapidly, but by the end of the 7-day feeding period, the biogas production had fallen back significantly although never to zero (data not shown). Within a week and among the reactors the daily fluctuations reached 40%, taking into account the entire test period the average fluctuations were 23 and 24 % for pig blood-fed and casein-fed reactors, respectively. The substrate remaining in the reactor could contribute to the subsequent biogas production hence it could lead to apparently overestimated biogas yield values in weeks 7-10 when maximum biogas production was attained (Figures 2, 3). The biogas yields were therefore determined in separate batch tests. In the case of casein 0.403 ± 0.004L CH_4_/g oDM while for pig blood 0.420 ± 0.003 L CH_4_/g oDM were measured under normal conditions. Although the methane contents of the biogas were 52 and 51% for casein and blood, respectively, the yields are significantly higher than yields from commonly used biogas substrates, e.g. animal manure (0.12 L CH_4_/g oDM), maize silage (0.29 L CH_4_/g oDM), or sugar beet silage (0.25 L CH_4_/g oDM) [45].

**Figure 4:**
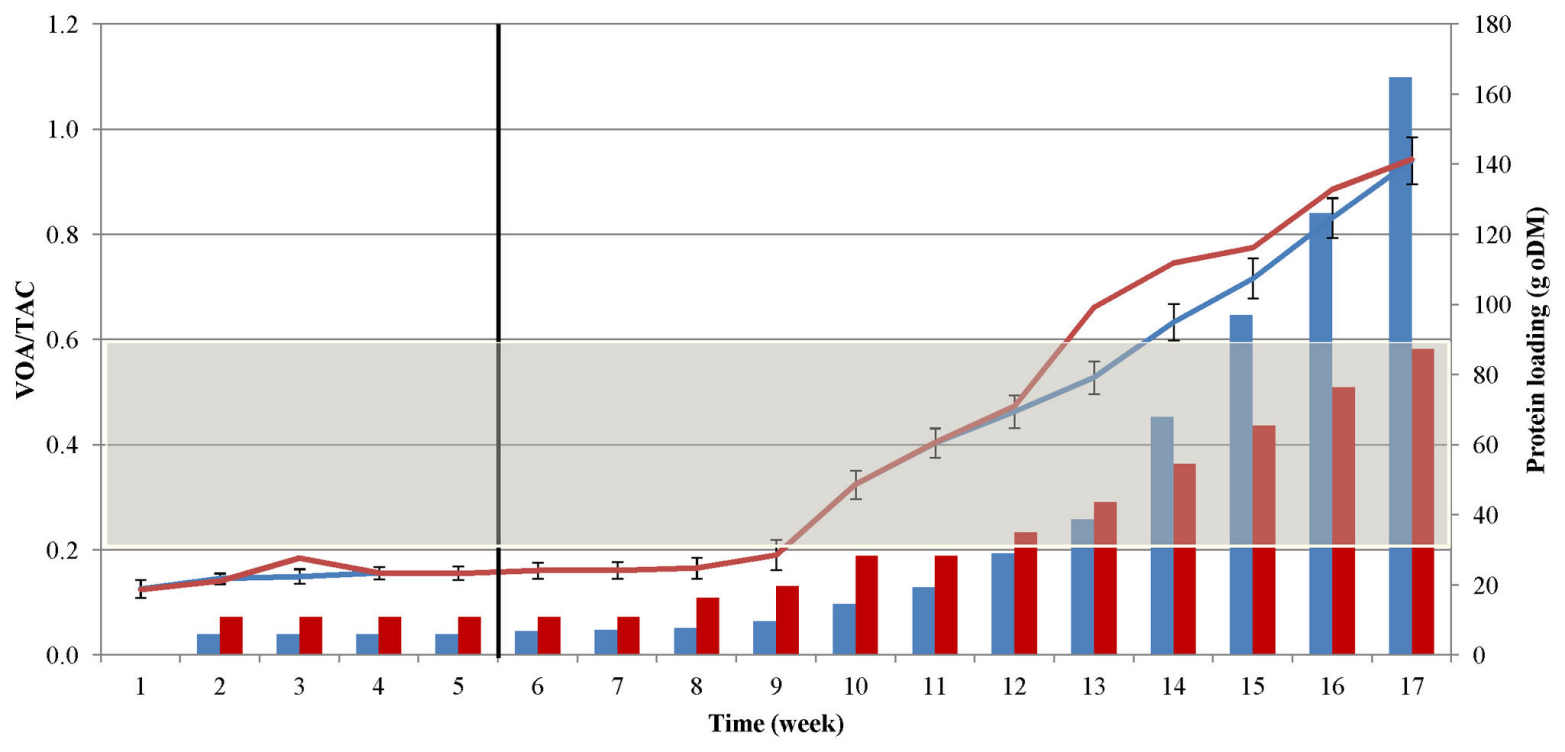
Changes in the VOAs/TAC values during the AD of casein (blue line) and blood (red line). The protein loads administered at weekly intervals are indicated by the blue (casein) and red (blood) columns. The vertical black line denotes the end of the adaptation period, i.e. feeding with a constant, low protein dose. The gray area indicates the VOAs/TAC range under optimal operational conditions.

In the case of pig blood, the situation was somewhat similar to that with casein, but the process did not collapse as quickly as in the case of casein. Besides fibrinogen, the precipitated blood protein sample contains important minerals, which could contribute to stabilization of the system. Moreover, the rate of increase of the protein dose was not as high as in the case of casein (see Figures 1 and 4), and therefore the microbes were exposed to less stressful conditions. Optimization of the adaptation and feeding strategy may further improve the process stability.

From the classical equation developed by Symons and Buswell [46] the theoretical biogas potential is 0.5 L CH_4_ from 1 g “average” protein oDM. During casein fermentation the mean CH_4_ content was 53% and the average biogas yield was 0.462 NL/g oDM; during AD of blood protein the average CH_4_ concentration was 54% and the average biogas yield was 0.476 NL/g oDM. As indicated earlier the daily and weekly biogas production fluctuated significantly. The benefit of the fed-batch operational mode is in the accumulation of digestible substrate during the adaptation phase. This finding suggests that fed-batch feeding may be the recommended approach when substantial change in substrate and concomitant microbial community is anticipated. By taking into account the entire experimental period the average yields of 0.25 NL CH_4_/g casein oDM and 0.26 NL CH_4_/g blood protein oDM were estimated. It is clear from Figures 2 and 3 that the system operated under suboptimal conditions in the adaptation period and from week 9-12, when symptoms of process failure started to manifest themselves. This explains the difference between the average biogas yields in these experiments and the biogas productivity determined in the batch fermentations. 

The data sets were subjected to Pearson statistical correlation analysis [47] and a strong correlation between protein loading and biogas production was found. The Pearson coefficients during the first 9 weeks were 0.7408 and 0.7478 for casein and pig blood, respectively. An even stronger negative correlation between the cumulative protein input and biogas production was observed during the “protein overload” phase, i.e. in weeks 11-15 in case of casein (the Pearson coefficient r= -0.9400) and weeks 9-16 in case of blood protein (r= -0.9749). The negative signs of the Peason coefficient clearly indicate an unbalanced operation of the system.

### Changes in the ratio VOAs/TAC during the AD of protein-rich substrates

The ratio of the volatile organic acids (VOAs) and total alkaline capacity (TAC) was proposed by Nordmann [48] and formulated by McGhee [49] as an appropriate measure of the functional stability of the AD process. A VOAs/TAC ratio below 0.2 means that the reactor needs feeding, whereas at a ratio ≥0.6 the biomass input is excessive and the process is out of balance. During adaptation to the protein substrate in our study, the microbes were supplied with only a limiting amount of substrate, which could serve as an incentive for them to adjust their metabolism to the unusual substance. As a test of the capacity of the system, the feeding intensity was increased until biogas evolution ceased due to failure of the process. Signs of the collapse of biogas production began to accumulate from week 12-13; the levels of VFAs increased, the specific gas production declined, and the ratio of VOAs/TAC approached and then exceeded the critical value of 0.6 (Figure 4).

The ratio VOAs/TAC proved to be a reliable indicator of process stability in our case. In this context, it should be noted that the pH in the reactor was monitored continuously and did not display any substantial change. It fluctuated between 8.01 and 8.32 and pH adjustment was therefore not necessary. During protein degradation, organic acids and ammonia were produced simultaneously and approximately equivalently, thereby balancing the pH.

### Accumulation of VFAs in protein-fed anaerobic reactors

**Figure 5:**
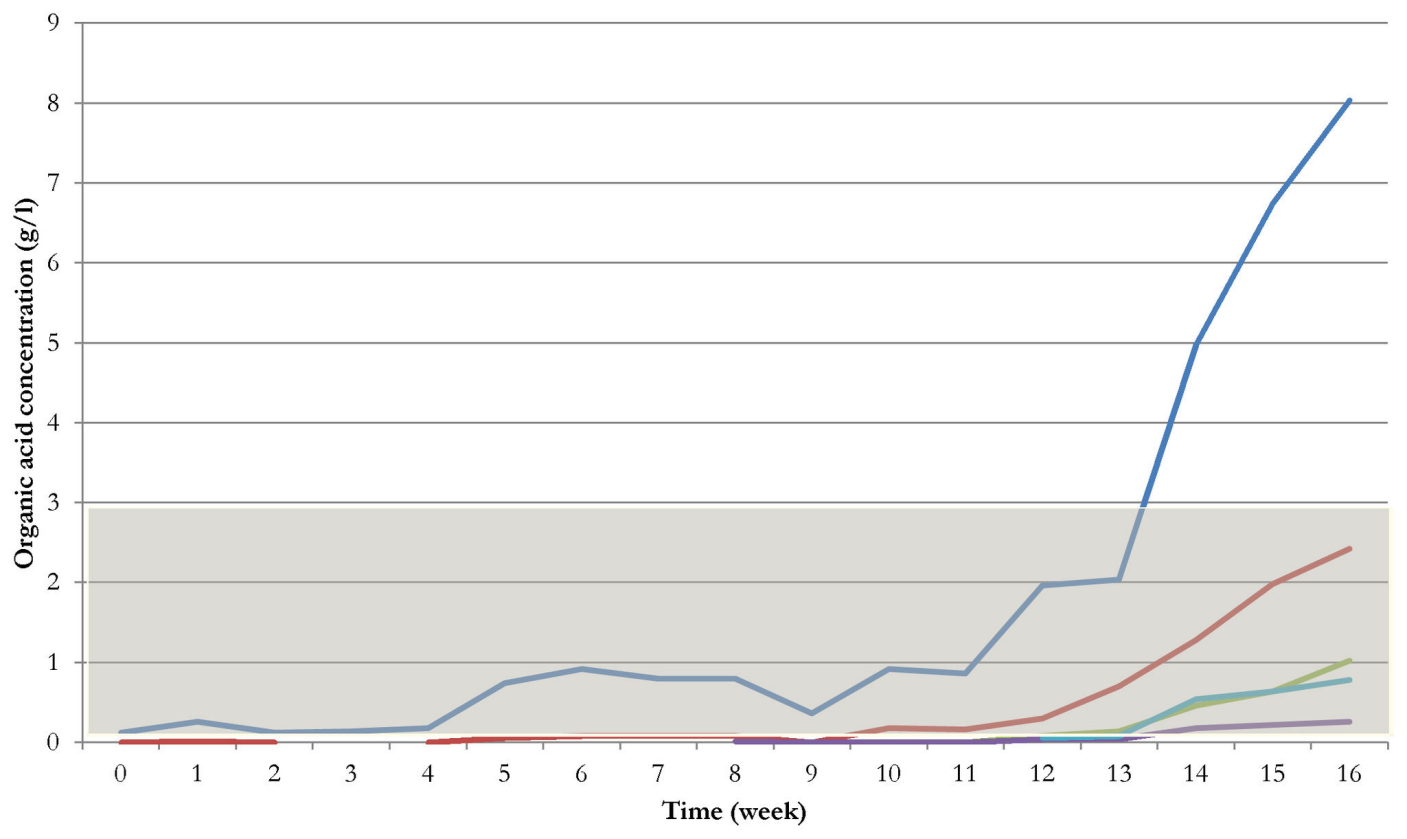
Changes in the volatile fatty acid accumulation during the AD of casein. The colors indicate the acetate (blue), propionate (red), isobutyrate (green), butyrate (violet) and isovalerate (light blue) concentrations. The gray area illustrates the acetate concentration range under optimal operational conditions. The vertical black line denotes the end of the adaptation period, i.e. feeding with a constant, low protein dose (see Figures 1 and 4).

During the first 4 or 5 weeks of the adaptation phase, the same, low level of protein substrate was supplied for the microbes. Before the weekly protein dose was increased, the level of biogas production continuously rose and low concentrations of VFAs were observed. At this stage, the protease activity did not change significantly (Figure 1). The acetate concentration reached the critical limit of 3 g/L in week 12-13 and the other signature organic acids too started to accumulate. At the same time, the biogas yield began to decline, probably due to the dwindling methanogenic activity. Since the pH did not change, a direct VFA blocking of the process or NH_3_ accumulation may have been responsible for this inhibition. The accumulation of VFAs is illustrated in Figure 5 for casein as substrate. The AD of pig blood displayed essentially the same behavior (data not shown). Overall, the results suggest that, in the early stages of fermentation, the availability of proteases was probably the limiting factor, but overloading with protein was also deleterious due to the accumulation of metabolites.

### Changes in concentrations of NH_3_ / NH_4_^+^and H_2_S during adaptation to the protein substrate

**Figure 6:**
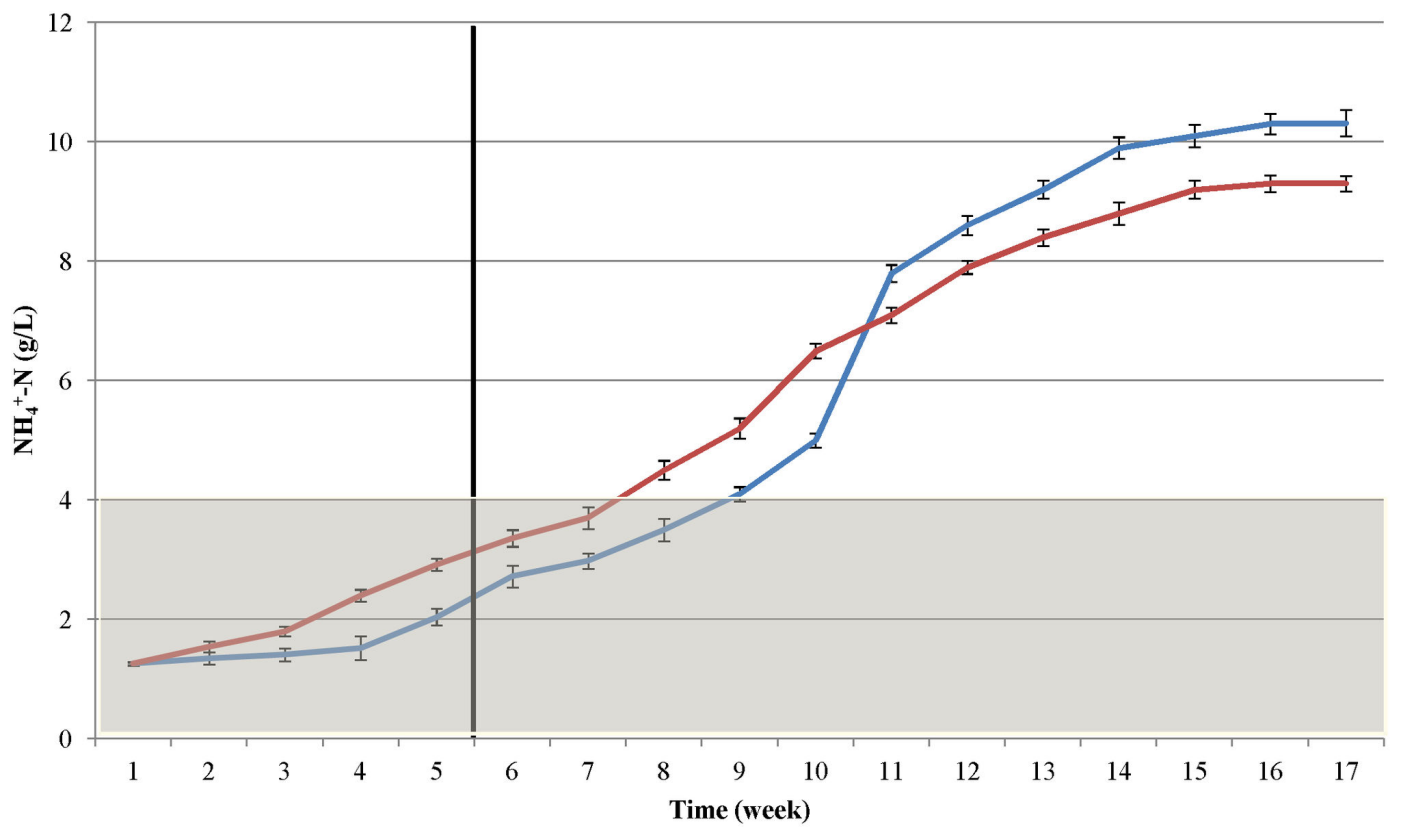
Accumulation of ammonium during the AD of casein (blue line) and pig blood (red line). The gray area indicates the ammonium-nitrogen concentration range under optimal operational conditions. The vertical black line denotes the end of the adaptation period, i.e. feeding with a constant, low protein dose.

The dissolved NH_4_^+^ level gradually increased during the course of the experiment (Figure 6). This is not greatly surprising as the bacteria received a protein-only feed and one of the decomposition products is ammonia. The NH_4_^+^-N concentration started to increase even during the acclimation period, and rose above the critical level of 4 g/L [33,37] before the biogas production reached its maximum. The system demonstrated stable operation and increasing biogas productivity up to 7-8 g NH_4_^+^-N/L. There was no striking difference between the casein and pig blood substrates as regards the generation of ammonia, though it is noteworthy that the patterns closely resembled those of the protein dosage (see Figures 1 and 4), but did not follow the trend of VFA accumulation (Figure 5).

The gradual build-up of H_2_S, which was accompanied by a growth on the protein-rich substrates, reached 1,500-1,800 ppm at the time of a protein overload (data not shown).

### Comparison of the protein substrates

**Figure 7:**
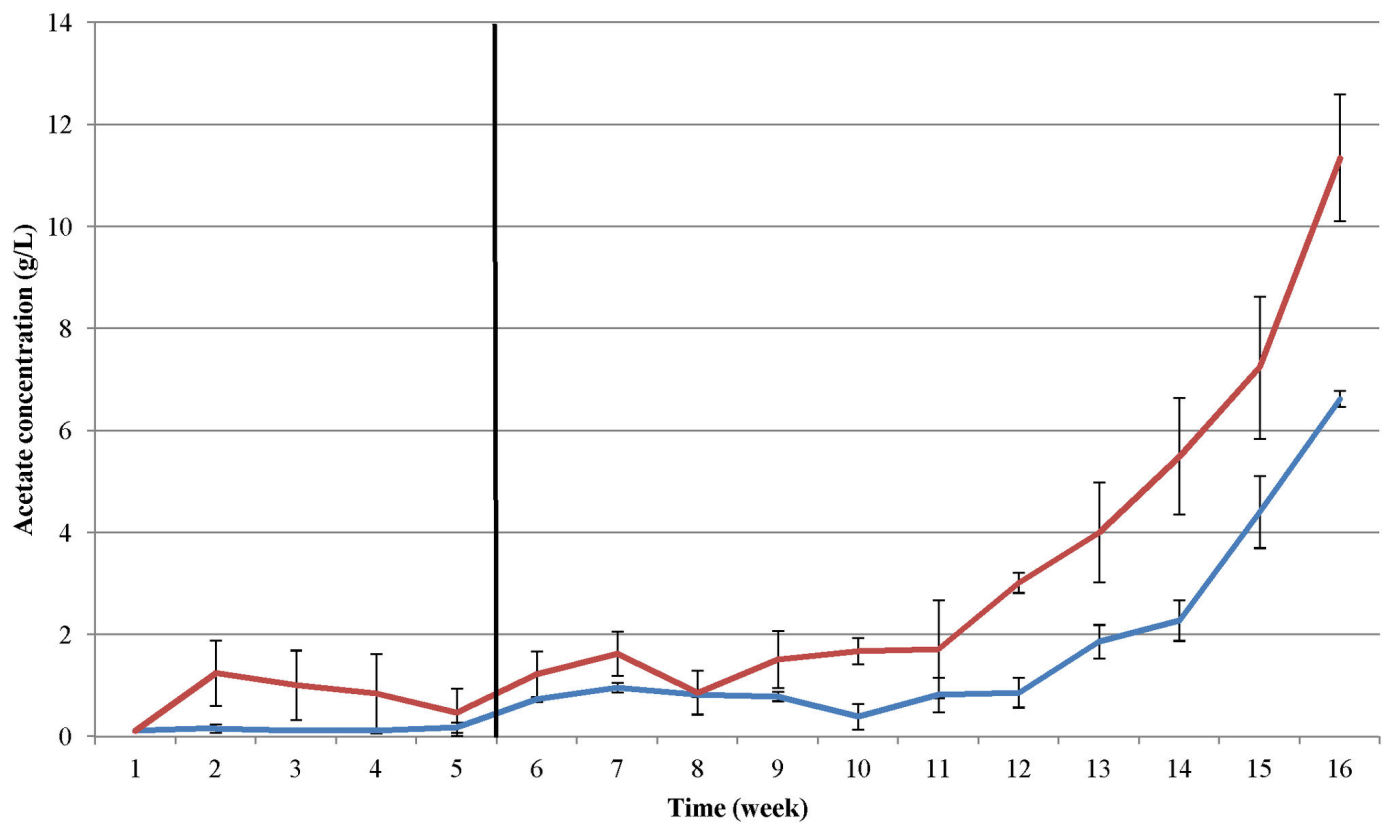
Comparison of acetate accumulation during growth on casein (blue line) or on pig blood (red line) in fed-batch AD. The vertical black line indicates the end of the adaptation period, i.e. feeding with a constant, low protein dose.

The changes in the acetate concentrations produced from the two proteinaceous substrates are illustrated in Figure 7. Before the addition of the first protein-rich dose, a stable process (with low acetate levels) was observed in both cases. The switch to exclusive protein feeding did not result in a marked increase in acetate concentration for several weeks. Only about 3 months after the continuously increasing weekly protein substrate load had been instigated was a noticeable change observed in the acetate concentration. In week 12, the production of acetate through the fermentation of the pig blood started to exceed the critical concentration of 3 mg/mL. The casein fermentation reached the acetate threshold of 3 mg/mL in week 15. The rate of increase of the protein dosage was lower in the case of the pig blood (Figures 1 and 4), and was accompanied by more efficient biogas production and higher stability against process failure (Figures 2 and 3) relative to casein, but the trend was different when the rates of acetate accumulation were compared. A consideration of this observation together with the accumulation of ammonia suggests that, although both systems tolerated a fairly high level of ammonia, inhibition by ammonia was eventually the most likely reason for the process failure under protein overload conditions in the fed-batch reactors.

### Scaling-up of biogas production from a protein-rich biomass

**Figure 8:**
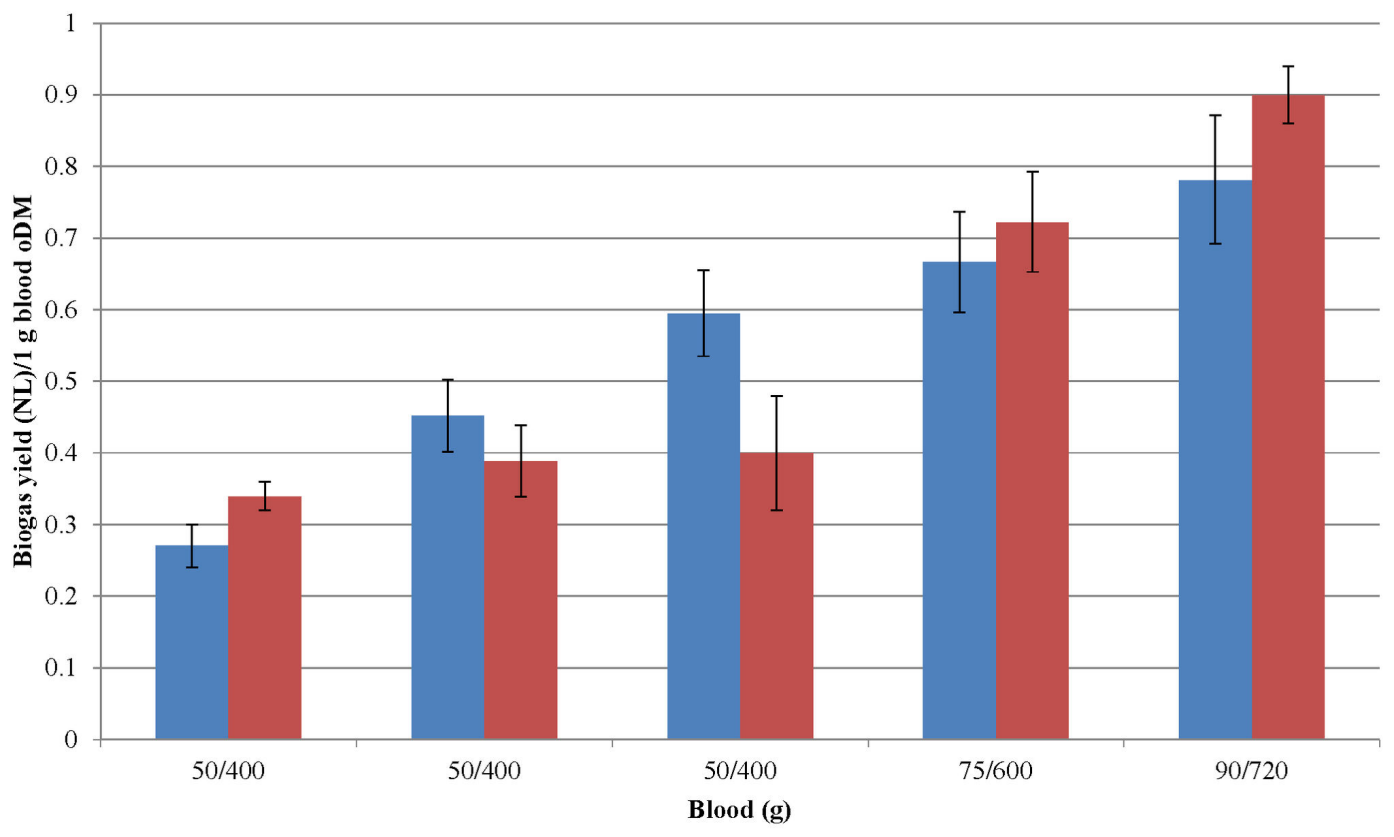
Scaling-up of AD of blood protein. Weekly average specific biogas yields were determined in completely stirred tank reactors of 5 L (blue columns) or 50 L (red columns). The X-axis indicates the weekly substrate doses for the 5 L (first number) and 50 L (second number) reactors. Note that both reactors were fed with the same amount of substrate for 3 weeks and the weekly dose was raised afterwards.

All of the experiments presented so far were carried out in reactors with a working volume of 5 L. The possibility of the scaling-up of the process was next investigated. For this purpose, reactors with a very similar design, but with a working volume of 50 L were constructed. The design and placement within the vessel of the impeller, the feeding and effluent disposal arrangements and the monitoring sensors were also similar. The substrate in these tests was precipitated pig blood protein. It has been widely observed in practice that scaling-up is generally accompanied by lower yield and efficiency [50]. In our case, however, the larger reactors performed at least as good as the smaller ones although after a somewhat slower adaptation (Figure 8). Efficient biogas production from pig blood was accomplished in both reactor sizes, indicating that major obstacles appear to be unlikely if such protein-rich substrates are applied in an even larger fermentation volume. This is encouraging, although clearly in no way sufficient from the aspect of direct large-scale applications.

### Stability of biogas production from protein-rich substrates in time

**Figure 9:**
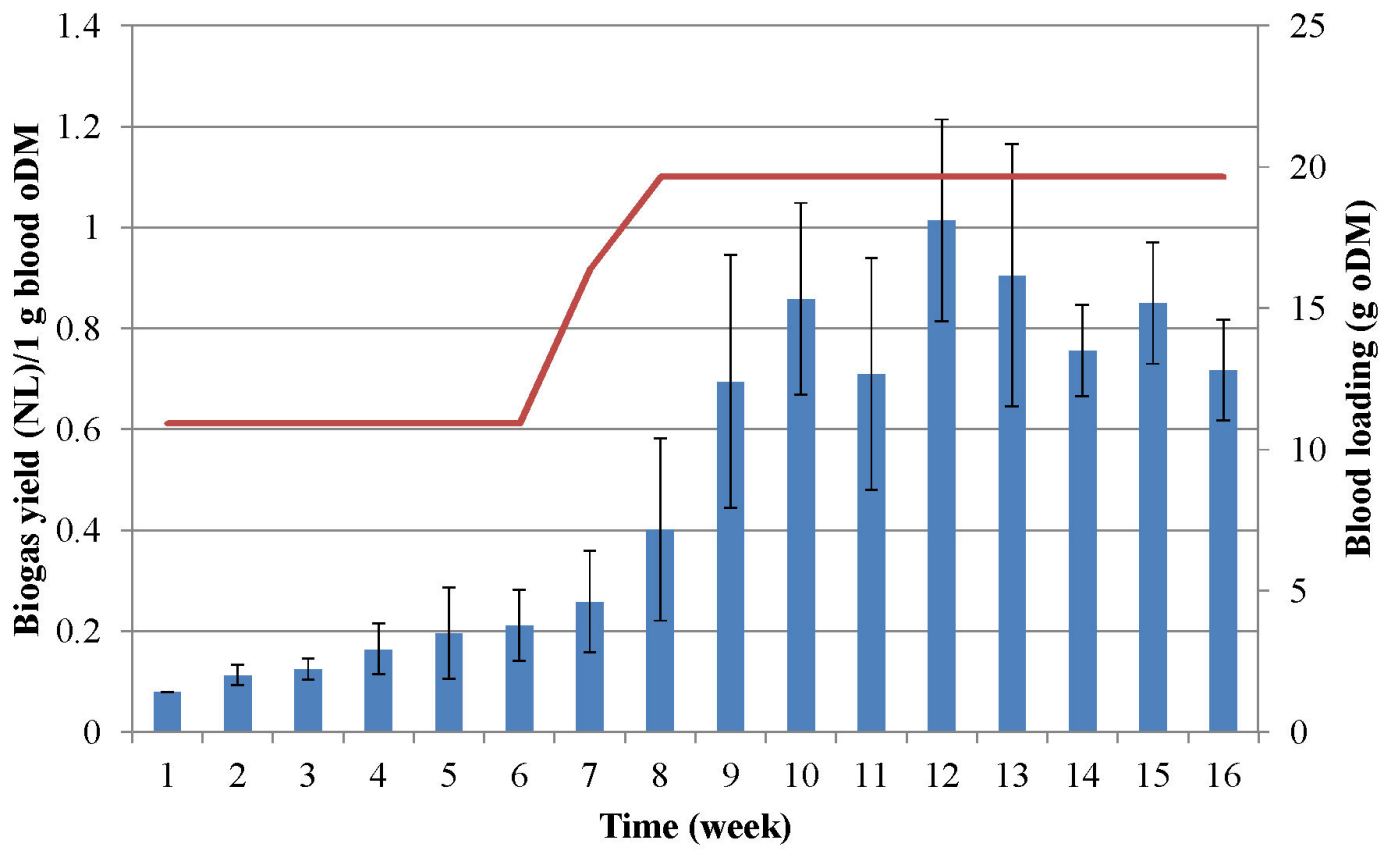
Testing of sustainability of biogas production from pig blood. The weekly average specific biogas production is indicated by the blue columns, and the blood protein dosing is shown by the red line.

In order to test the sustainability of biogas production from a protein-only substrate, the same weekly dose of pig blood protein was administered into the fed-batch 5 L AD reactors. Previous experiments (Figures 3 and 4) established that maximum biogas yield was generated from 18-20 g oDM of pig blood protein per week. This quantity of protein was dispensed weekly for 2 months (weeks 8-16 in Figure 9) after an adaptation period of 5 weeks and a gradual elevation of the protein dose during the subsequent 3 weeks. 

The pH in the reactor varied between 8.0 and 8.4, no pH adjustment was necessary. The ratio VOAs/TAC did not change significantly either, the value ranging between 0.13 and 0.20 throughout these experiments. The CH_4_ content of the gas remained practically constant at 54-58%. The H_2_S concentration in the evolved gas, however, increased from an initial 300 ppm (week 2) to 1,600 ppm (week 16), which is considerable. The NH_4_^+^-N concentration also rose during the sustainability test, although not as markedly as that of H_2_S, and it stabilized at 8-10 g/L in weeks 8-16. 

During the period of constant feeding (weeks 9-16 in Figure 9) the average CH_4_ content of biogas was 55% therefore the average CH_4_ yield was 0.447 L CH_4_/ g oDM. This compares well with the yield of 0.420 L CH_4_/ g oDM determined in batch tests and indicates that a well-adapted microbial community is able to convert the protein-rich substrate with 89% conversion efficiency relative to the theoretical maximum. A biogas production efficacy this high is remarkable particularly in the case of biogas production from practically a monosubstrate. The application of fed-batch fermentation technology may have contributed to this result as some portion of the substrate stayed in the reactor for an extended period of time.

### Changes in the microbial community composition upon adaptation to casein

During the fed-batch fermentations, the composition of the microbial community was determined 4 times in the case of the AD of casein: at the start of the adaptation (week 0), after the adaptation period (week 5), when the system was working at full capacity (week 9), and toward the end of the process (week 12), when signs of process failure were emerging. The structure of the methanogenic community was determined by using a highly parallel SOLiD^®^ (Sequencing by Oligo Ligation and Detection) next-generation DNA sequencing approach [11]. 

The distribution of the species in the biogas-producing microbial community at the beginning of the experiments (week 0) was very similar to those revealed in earlier studies on the microbial community composition in the conventional AD process with animal manure and maize silage as substrates [11,49-51]. This was to be expected, as the inocula used to start our fermenters originated from the AD of pig manure and maize silage. The results obtained at this stage may therefore be regarded as an internal control validating the metagenome sequencing approach [11]. 

Several characteristic alterations were observed relative to the initial microbial composition (week 0) as a result of the adaptation process (week 5). As concerns the overall experimental period, the greatest alterations within the Bacteria domain was demonstrated by the Firmicutes and Proteobacteria phyla. The classes Clostridia, Bacilli and Gamma-proteobacteria constituted the majority of the Bacteria in the biogas digester. 

**Figure 10:**
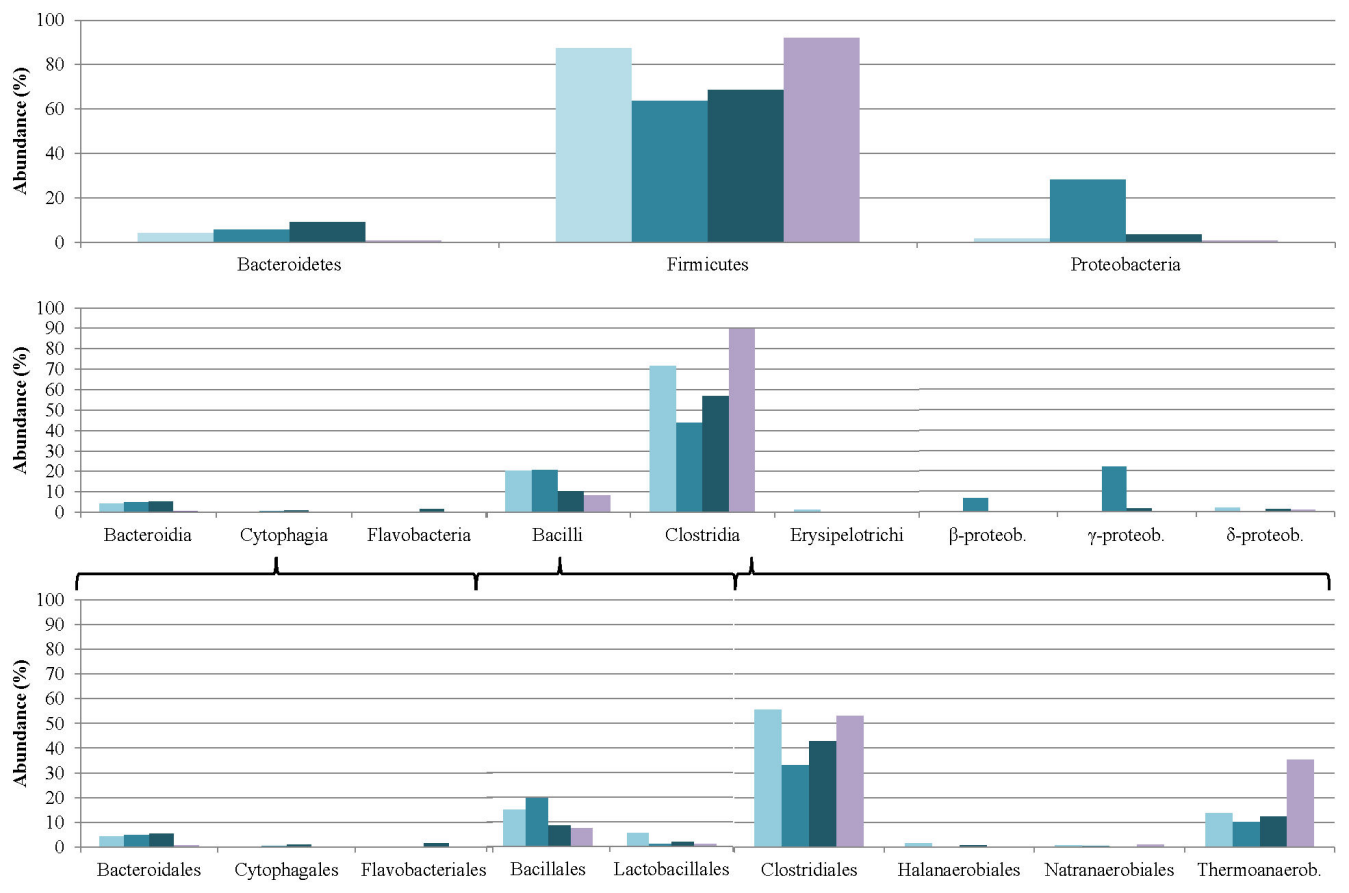
Changes induced in the Bacteria microbial community composition by adaptation to casein as sole carbon source. The relative abundances of the taxonomic groups were determined in week 0 (light-blue columns), week 5 (medium-blue columns), week 9 (dark-blue columns) and week 12 (purple columns).

By the end of the adaptation experiments, as expectable, protein degrading species and those that utilize amino acids or other nitrogen-rich compounds dominated the community. Within the Bacilli class, the members of the Bacillales and Lactobacillales orders became more abundant. In the Clostridia class, the Clostridiales and Thermoanaerobacterales orders displayed very pronounced changes (Figure 10). Only traces of Natranaerobiales were detected in the non-adapted community, and a perceivable increase in their number was observed relative to their initial abundance. The abundance of cellulose or other carbohydrate-degrading bacteria fell significantly; indeed some of them had vanished by the end of the protein fermentation. 

Similarly to earlier related findings [11,51-53], representatives of the Firmicutes phylum constituted the overwhelming majority of the total abundance in the original inoculum (week 0). As the adaptation to the protein-only substrate progressed (week 5), a transient decrease in the abundance of Firmicutes was accompanied by an elevation in the number of Proteobacteria. At the peak of biogas productivity (week 9), the Firmicutes regained momentum and they reached essentially the same abundance when the system failure started to emerge (week 12). Similar trends were observed in the Clostridia class and Clostridiales order, whereas the Bacilli class and Bacillales order exhibited a continuous loss in representation. This trend was probably due to the reduction in the polysaccharide-degrading species, which lost their importance during protein feeding. 

In parallel with the partial reduction of the polysaccharide-consuming members of the microbial community, the protein-degrading bacteria started to take over as the acclimation to the protein-rich substrates progressed. Some of the outstanding representatives of this group increased their relative abundances considerably (Table 1). A surprising exception was *Candidatus* Cloacamonas *acidaminovorans*, which was practically eliminated from an abundance of 2.0% to 0.0%.

**Table 1:** The most significant changes at a strain level in the abundance of Bacteria (%) during casein fermentation.

**Strain**	**Weeks of fermentation**			
	**0**	**5**	**9**	**12**
***Caldanaerobacter subterraneus***	0.8	0.9	2.5	5.3
***Clostridium sticklandii***	0.3	0.3	0.3	1.5
***Thermoanaerobacter pseudethanolicus***	0.4	0.4	0.7	2.3
***Alkaliphilus metalliredigens***	1.0	1.3	3.9	6.5
***Alkaliphilus oremlandii***	0.8	0.8	2.6	3.90
***Candidatus Cloacamonas acidaminovorans***	2.0	0.6	0.0	0.0

*Caldanaerobacter subterraneus*, which produces L-alanine as a major end-product, requires yeast extract or bio-trypticase for its growth [54]. *Clostridium sticklandii* degrades amino acids in a preferred sequential way. The organism prefers threonine, arginine, serine, cysteine, proline and glycine as carbon and energy sources and excretes glutamate, aspartate and alanine. Energy conservation is primarily obtained by substrate-level phosphorylation in fermentative pathways. An unusual feature of *Cl. sticklandii* is the presence of genes coding for the enzymes involved in two distinct CO_2_-fixation pathways; this bacterium harbors both the glycine synthase/glycine reductase and the Wood-Ljungdahl pathways [56]. *Thermoanaerobacter pseudethanolicus* cells reduce thiosulfate to H_2_S. They can metabolize various carbohydrates, including xylose, cellobiose, starch, glucose, maltose and sucrose, and they can also utilize proteinaceous substrates. No growth was observed on a CO_2_/H_2_ mixture [57], and H_2_ consumption is therefore unlikely. *Alkaliphilus metalliredigens* (QYMF strain) grows with the utilization of Fe(III)-citrate, Fe(III)-EDTA, Co(III)-EDTA or Cr(VI) as electron acceptor, and yeast extract and lactate could serve as electron donor [58]. Interestingly, *A. metalliredigens* was identified earlier as a major player in the methanogenic consortia [11]. Although it may not be trivial to explain the occurrence of a metal-reducing bacterium in an anaerobic biogas-producing community, it should be noted that these bacteria also possess highly active [FeFe]-hydrogenases [59]. Metal-reducing organisms, such as *Geobacter metallireducens*, have been implicated in direct electron transfer between the syntrophic partners within the methanogenic community [60]. Serving as electron sinks, they can inhibit the activity of sulfate-reducing bacteria and methanogenesis [61], but in the AD of pig manure they facilitated biogas formation [62]. *Alkaliphilus oremlandii* is a strict anaerobe, which ferments lactate via the acrylate pathway, and also utilizes fructose and glycerol. It additionally has a respiratory capability, being able to use arsenate and thiosulfate as terminal electron acceptors with acetate, pyruvate, formate, lactate, fumarate, glycerol or fructose as electron donor [63]. Its ability to grow on protein substrate has not been recognized previously. *Candidatus* Cloacamonas *acidaminovorans* can oxidatively deaminate amino acids for use as growth substrates. It obtains most of its carbon and energy from the fermentation of amino acids. Amino acids are converted to pyruvate, a central metabolic intermediate. It is a fermentative H_2_ producer, containing an [FeFe]-hydrogenase, which is an indication of a syntrophic metabolism. *C. Cc. acidaminovorans* cannot produce polyamines and a number of cofactors: thiamine, biotin, lipoic acid, pyrrolo-quinolinequinone, coenzyme B12, folic acid, pyridoxine and heme [53,55]. All these ingredients must be obtained from the environment, which may explain why it cannot tolerate a drastic change in substrate composition and cannot grow on protein monosubstrates, despite their beneficial properties. The disappearance of *C. Cc. acidaminovorans* exemplifies the fact that a multitude of appropriate conditions are required for a strain to become a stable member of the methanogenic community in response to changes in substrate composition. 

**Figure 11:**
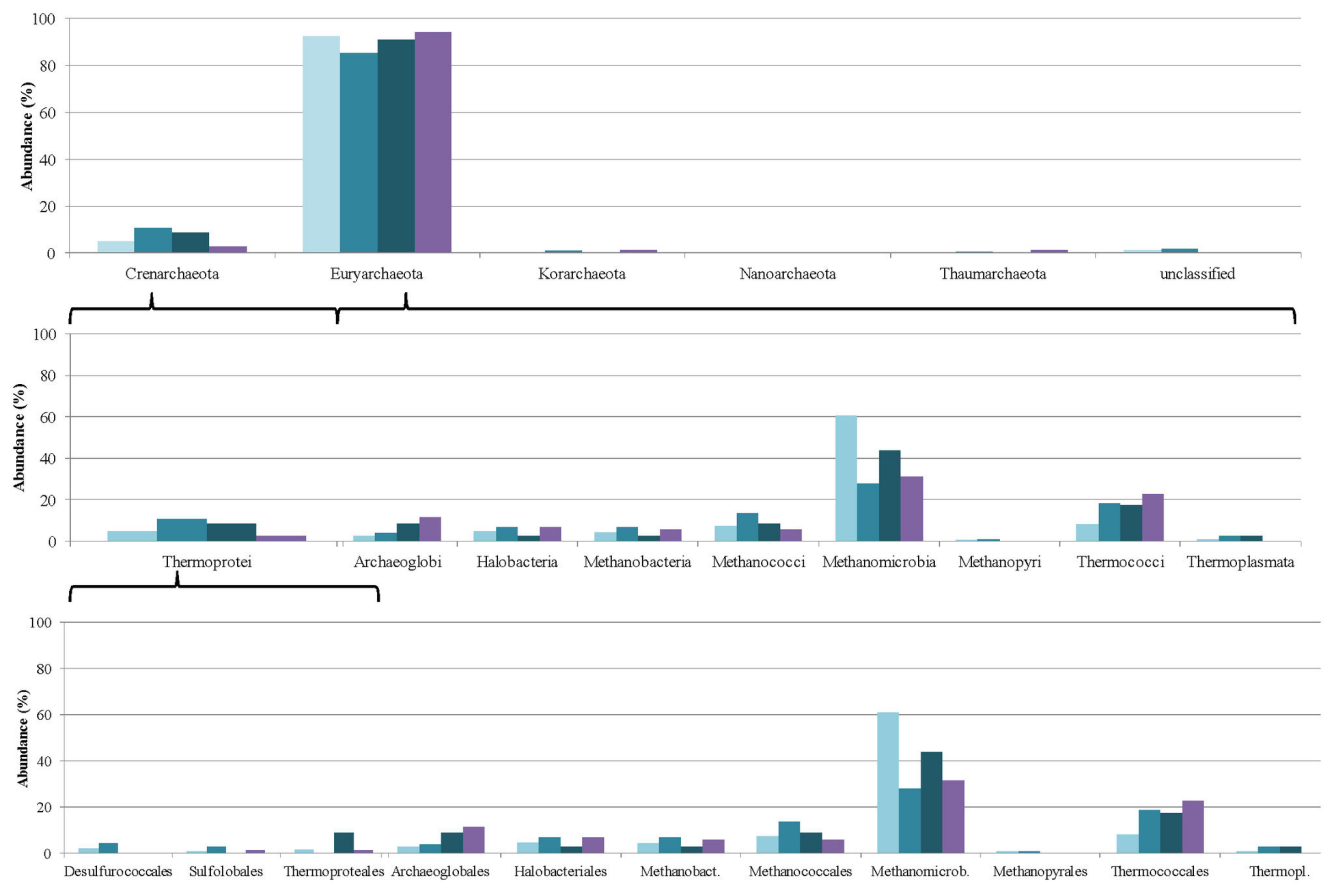
Changes induced in the Archaea microbial community composition by adaptation to casein as sole carbon source. The relative abundances of the taxonomic groups were determined in week 0 (lightblue columns), week 5 (medium-blue columns), week 9 (dark-blue columns) and week 12 (purple columns).

The Archaea community is represented at a rather low level, i.e. <10% of the total abundance of microbes in the community. Moreover, they display a diverse distribution (Figure 11), almost all archaeal phylogenetic groups being represented.

The Euryarcheota dominate in the archaeal community. Within this phylum, the members of the Methanomicrobia class appear to be most abundant, followed by the Thermococci and Methanococci. A similar pattern of rearrangements was observed among the Archaea as in the Bacteria domain, i.e. during the adaptation period the Methanomicrobia first decreased in abundance (week 5), and then recovered (week 12), just as the Clostridia did (Figure 10). The Methanococci behaved similarly to the Bacilli among the Bacteria. The Thermococci class demonstrated a distinct response to casein as substrate: they were stimulated by the substrate and increased their relative representation in the community. Nevertheless, because of the relatively small number of Archaea in the community, an in-depth analysis of their distribution at higher resolution involves a high level of ambiguity. A T-RFLP study on changes in the archaeal community in samples taken from the same fermentations at week 1 and week 5 of the adaptation process revealed that essentially the same strains could be detected by both approaches. However, the relative abundances displayed a somewhat distinct pattern [64]. This variation could be explained by the different specificity levels being biased by the PCR steps in both approaches.

### Changes in the microbial community composition upon adaptation to pig blood protein

Some of the alterations observed in the composition of the biogas-producing microbial community when precipitated pig blood protein was used as monosubstrate in the AD process were very similar to the results discussed above as regards, casein. 

**Figure 12:**
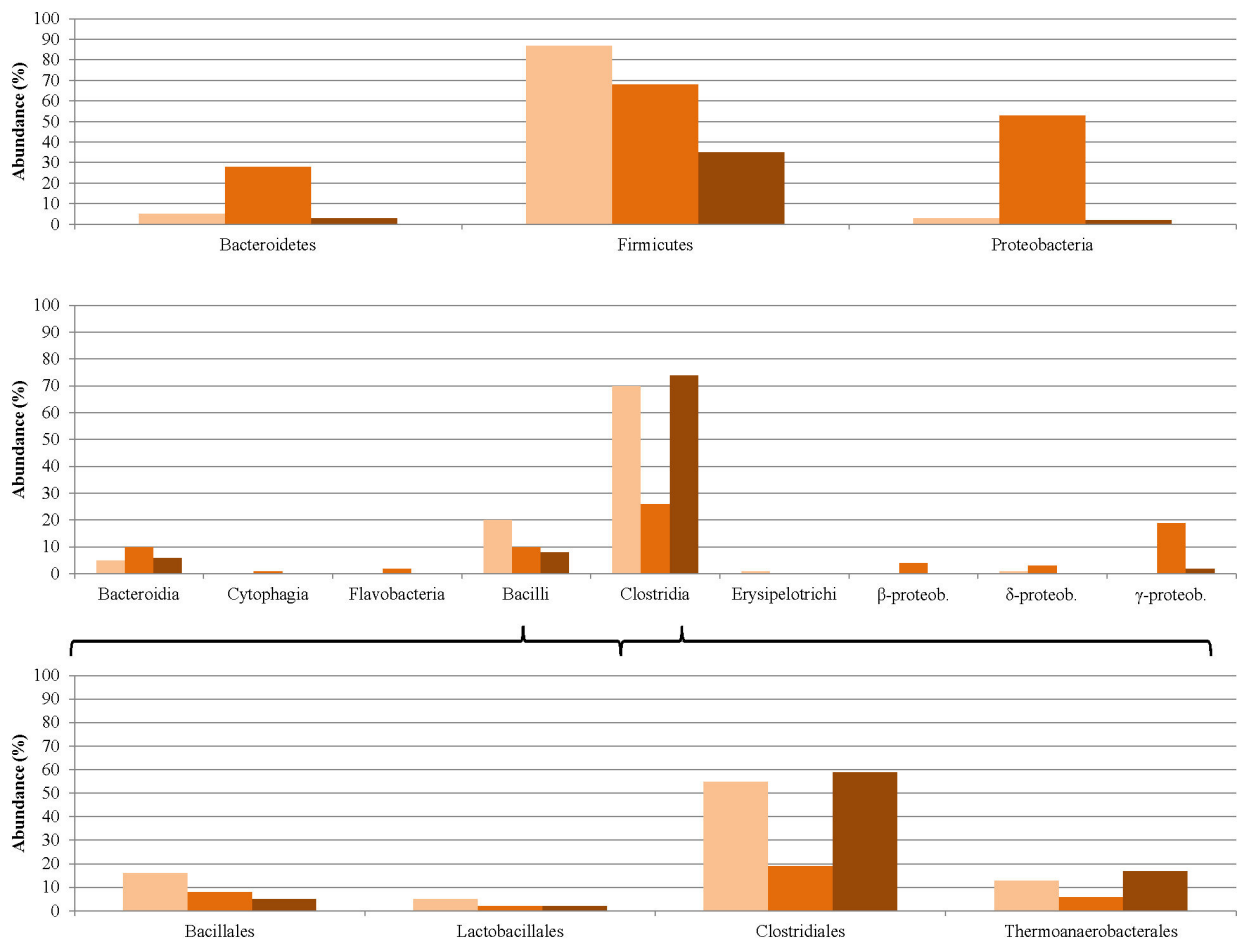
Changes induced in the Bacteria microbial community composition by adaptation to pig blood protein as sole carbon source. The relative abundances of the taxonomic groups were determined in week 0 (light-brown columns), week 8 (medium-brown columns) and week 12 (dark-brown columns).

In this case too the Firmicutes phylum dominated the bacterial domain, although the Bacteroidetes and Proteobacteria were also characterized by a considerable representation, particularly in week 8, i.e. at the peak of biogas-producing activity (Figures 12 and 3). Although their relative number decreased during the experiment, at the taxonomic level of classes and orders the same tendency was seen as in the case of casein as substrate. 

The most pronounced changes at a strain level were observed *Alkaliphilus metalliredigens* and *A. oremlandii*, which were present in high abundance similarly as in the AD of casein. Complementing the common acclimation picture the number of *C. Cc. acidaminovorans* decreased to practically zero, indicating that this strain could not adapt well to the protein substrate, or that some component vital for its growth was not present in these monosubstrates (Table 2). Several other differences in the responses to the substrates were probably a result of the differences in minor components between the milk by-product and the slaughterhouse waste blood fraction (see Table 3). 

**Table 2:** The most significant changes at a strain level in the abundance of Bacteria (%) during fermentation of pig blood.

**Strain**	**Weeks**		
	**0**	**8**	**12**
***Alkaliphilus metalliredigens***	1.0	1.9	8.7
***Alkaliphilus oremlandii***	0.5	1.1	5.7
***Dethiosulfovibrio peptidovorans***	0.2	1.1	0.4
***Anaerobaculum hydrogeniformans***	0.1	4.7	0
***Candidatus Cloacamonas acidaminovorans***	2.0	2.0	0

**Table 3:** The properties of the substrates used in this study.

**Property**	**Unit**	**Casein**	**Blood**
**Organic total solids (oDM)**	% of TS	59.83	95.79
**Total solids (TS)**	%	97.12	23.32
**Water content**	%	2.88	76.68
**Density**	kg/m^3^	1.479	1.058
**Total nitrogen**	g N/kg	83.85	52.66
**Total carbon**	g C/kg	290.8	168.7
**C/N ration**		3.5	3.2
**Lipids**	% of oDM	<1	0.3
**Proteins**	% of oDM	82.8	94.4
**Carbohydrates**	% of oDM	<2.4	5.3

*Dethiosulfovibrio peptidovorans* proliferated effectively on the pig blood substrate. This bacterium utilizes peptides and amino acids, but cannot live on sugars or fatty acids. It ferments serine, histidine and casamino acids, whereas arginine, glutamate, leucine, isoleucine, alanine, valine, methionine and asparagine are utilized only in the presence of thiosulfate. The peptides are converted to acetate, isobutyrate, isovalerate, 2-methylbutyrate, H_2_ and CO_2_, and this strain thrives particularly vigorously during the intensive biogas production period, but its copiousness decreases later [65]. In this work the abundance changed in a similar fashion in the case of *Anaerobaculum hydrogeniformans*, which demonstrated a clear preference for pig blood until the signs of process failure started to manifest. These examples reveal that the various strains respond to the stress conditions in different ways as concerns the accumulation of NH_4_^+^ and H_2_S. It should also be noted that the kinetics of biogas evolution differed somewhat with the two substrates. Higher resolution mapping of the fluctuations within the community should provide a more thorough understanding of these phenomena. 

**Figure 13:**
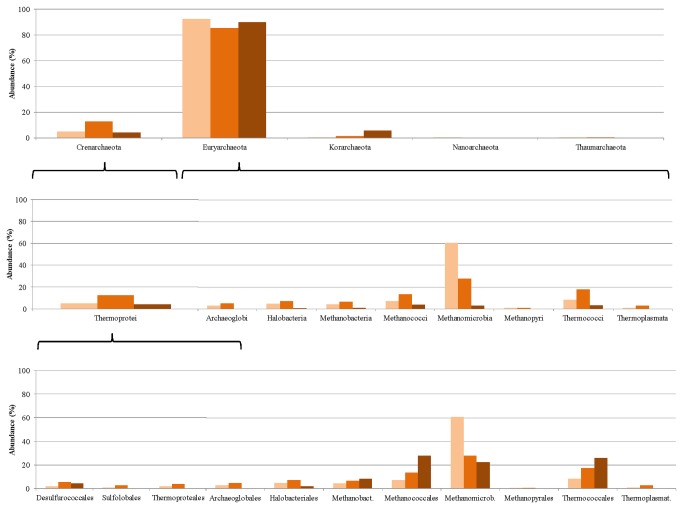
Changes induced in the Archaea microbial community composition by adaptation to pig blood as sole carbon source. The relative abundances of the taxonomic groups were determined on week 0 (light-brown columns), week 8 (medium-brown columns) and week 12 (dark-brown columns).

The distribution of the Archaea at the phylum level was similar to that for the casein-fed digesters (Figure 13).

The tendencies, however, indicated a mixed picture. The relative abundance of the Methanomicrobiales decreased and that of the Thermococcales increased as in the case of casein as substrate. The number of Methanococcales increased during the acclimation to the pig blood protein, whereas the opposite was observed when the community was producing biogas from casein.

In summary, the possibility of using protein-rich substrates with very low C/N ratios for biogas production in a fed-batch stirred tank reactor has been demonstrated. A prerequisite for the maintenance of the efficient AD of casein or pig blood was to subject the microbiological system to an adaptation process, the efficacy of which was followed by measuring the protease activity of the overall community. The AD of these proteinaceous substrates led to very high biogas production with biogas yields (in terms of oDM) significantly higher than those from maize silage or animal manure [45]. The process parameters indicated an imbalance in the event of protein overloading. The tendencies in the changes in the various indicators pointed to ammonia and H_2_S as factors determining the eventual collapse of the microbiological community when too much protein was fed into the reactors. Nonetheless, the system operated steadily with these protein-rich substrates below the overloading concentrations. Characteristic changes in the composition of the microbial community were detected through the use of next-generation sequencing, and the community structures were determined for both substrates at various stages of the process by using a metagenomic approach. Besides the common alterations in the microbial community structure that were attributed to the introduction of the proteinaceous substrates, specific responses to the particular substances were recognized. These findings promote an understanding of the dynamic changes within the community during the AD of organic wastes [66]. This may eventually lead to the introduction of the prevalent species and thereby the rational design of more efficient biogas-producing communities, which could shorten or eliminate the adaptation to shifting substrate composition.

## Methods

### Origin and composition of substrates

Casein (from bovine milk) was purchased from Sigma-Aldrich (CAS 9000-71-9). Pig blood was obtained from a local slaughterhouse (Smoked Sausage Ltd., Makó, Hungary), with permission to use it in the reported experiments, and the protein fraction was separated after coagulation at room temperature. The most important parameters of the substrates are summarized in Table 3.

### Determination of protease activity

Relative protease activity was determined by measuring the release of acid-soluble material from azocasein (Sigma). 200 µL of filtered (cellulose-acetate membrane, pore size: 0.2 µm, Whatman)sample was added to 50 µL of phosphate buffer (6.7×10^−2^ M, at pH 7.0) and 200 μL of 1% (w/v) azocasein. Following incubation at 37 °C for 1 hour, 700 μL of ice-cold 5% (w/v) trichloroacetic acid was added to stop proteolysis, with simultaneous vortexing. The sample was placed on ice for 10 min before centrifugation at 13,000 rpm for 10 min. The quantity of acid-soluble material in the supernatant was measured via the absorbance at 440 nm. 

### Measurement of biogas production and gas composition

The laboratory-scale 5 L and 50 L AD reactors have been described in details elsewhere [67] and were used in fed-batch operational mode in these experiments. The reactors were operated using pig manure and maize silage mixture [11] until operation and biogas production became stabilized prior to starting the protein feeding. All fermentations were carried out in triplicates [67]. Batch biogas yields were determined according to the VDI standard protocol [68]. The composition of the evolved biogas was measured by taking 250-L aliquots from the headspace and injecting them into a gas chromatograph (6890N Network GC System, Agilent Technologies) equipped with a 5 Å molecular sieve column (length 30 m, I.D. 0.53 megabore, film 25 m) and a thermal conductivity detector. Nitrogen was used as carrier gas.

### Determination of process parameters

#### pH

A Radelkis OP-211/2 digital pH-meter was used to measure the pH of samples. The pH-meter was calibrated before each determination with standard buffers of pH 2.00 and 7.05. 

#### VOAs/TAC

5 g of fermenter substrate was taken for the analysis and diluted to 20 g with distilled water. The subsequent titration process is fully automatic (Pronova FOS/TAC 2000 Version 812-09.2008). The results for VOAs, TAC and VOAs/TAC are displayed after a few minutes.

#### VFAs

Volatile fatty acids were determined by high-performance liquid chromatography (a Hitachi Elite instrument, equipped with an ICSep ICE-COREGEL 64H column and a refractive index detector L2490) using the following parameters: 0.05 M H_2_SO_4_ as solvent, a flow rate of 0.8 mL/min, a column temperature of 50 °C, and detector temperature of 41 °C.

#### NH_4_^+^-N

This was determined by the Merck Spectroquant Ammonium test (1.00683.0001). 

#### H_2_S

The H_2_S content of the evolved gas was measured with the Hydrogen sulfide 100/a test tube from Dräger (CH 29101).

#### oDM

The dry matter content was quantified by drying the biomass at 105 °C overnight and weighing the residue. Further heating of this residue at 550 °C in a furnace until its weight did not change yielded the organic total solid content.

#### C/N

To determine C/N, an Elementar Analyzer Vario MAX CN was employed. This works on the principle of catalytic tube combustion under an oxygen supply at high temperatures (combustion temperature: 900 °C, postcombustion temperature: 900 °C, reduction temperature: 830 °C, column temperature: 250 °C). The desired components were separated from each other with the aid of specific adsorption columns (containing Sicapent, in CN mode) and determined in succession with a thermal conductivity detector. Helium served as flushing and carrier gas.

### DNA extraction

2-mL liquid fermentation samples were collected for total-community DNA isolation by applying a cetyltrimethylammonium bromide-based DNA extraction buffer [67, 69, 70]. Cell lysis was carried out at 55 °C overnight. Phenol:chloroform (1:1) was used to extract contamination and the genomic DNA was precipitated with ethanol (90%). The DNA pellet was resuspended in 100 μL of Tris-EDTA buffer [71]. Its quantity was determined in a NanoDrop ND-1000 spectrophotometer (NanoDrop Technologies); the DNA purity was tested by agarose gelelectrophoresis. This method yielded a pure (A_260_/A_280_ ≥ 1.8) and sufficient amount of total DNA (200–800 ng/μL).

### DNA sequencing and data handling

Sequencing was performed with Life Tech’s SOLiD™ V4 sequencing platform. 30 million reads (average read length 50 nt) were generated for each sample. Primary data analysis was carried out with software provided by the supplier (base-calling). The 50 nucleotide reads were analyzed, quality values for each nucleotide were determined, and the reads were assembled into contigs through use of the CLC Bio Genomics Workbench 4.6 program. The de novo contig assembly was followed by MG-RAST analysis. Details of the bioinformatic evaluation and statistical analyses have been published previously [11]. The sequence data have been uploaded on the NCBI database, accession number SRS445535.
